# Retracted: microRNAs in hepatocellular carcinoma: carcinogenesis, progression, and therapeutic target

**DOI:** 10.1155/2021/1916343

**Published:** 2021-02-19

**Authors:** BioMed Research International

*BioMed Research International* has retracted the article titled “MicroRNAs in hepatocellular carcinoma: carcinogenesis, progression, and therapeutic target” [1] due to extensive text overlap with the following sources [2–4], which were not cited in the article:

- Callegari, E., Elamin, B. K., Sabbioni, S., Gramantieri, L., & Negrini, M., “Role of microRNAs in hepatocellular carcinoma: a clinical perspective.” *OncoTargets and therapy*, 2013, 6, 1167-78. https://doi.org/10.2147/OTT.S36161. [2]
- Le, XF., Merchant, O., Bast, R.C. et al., “The Roles of MicroRNAs in the Cancer Invasion-Metastasis Cascade”, *Cancer Microenvironment*, 2010, https://doi.org/10.1007/s12307-010-0037-4. [3]
- Jie Sun, Haiqi Lu, Xian Wang, and Hongchuan Jin, “MicroRNAs in Hepatocellular Carcinoma: Regulation, Function, and Clinical Implications,” *The Scientific World Journal*, vol. 2013, 924206, 2013, https://doi.org/10.1155/2013/924206. [4]

The authors did not provide a satisfactory response and the article is therefore being retracted due to this overlap, with the agreement of the Editorial Board. The authors do not agree to the retraction.

## References

[1] Hung C.-H., Chiu Y.-C., Chen C.-H., Hu T.-H. (2014). MicroRNAs in Hepatocellular Carcinoma: Carcinogenesis, Progression, and Therapeutic Target. *BioMed Research International*.

[2] Negrini M., Callegari E., Elamin, Gramantieri L., Sabbioni (2013). Role of microRNAs in hepatocellular carcinoma: a clinical perspective. *OncoTargets and therapy*.

[3] le X.-F., Merchant O., Bast R. C., Calin G. A. (2010). The Roles of MicroRNAs in the Cancer Invasion-Metastasis Cascade. *Cancer Microenvironment*.

[4] Sun J., Lu H., Wang X., Jin H. (2013). MicroRNAs in Hepatocellular Carcinoma: Regulation, Function, and Clinical Implications. *The Scientific World Journal*.

